# Availability of a sports dietitian may lead to improved performance and recovery of NCAA division I baseball athletes

**DOI:** 10.1186/s12970-017-0187-6

**Published:** 2017-08-10

**Authors:** Michael V. Hull, Jonathan Neddo, Andrew R. Jagim, Jonathan M. Oliver, Mike Greenwood, Margaret T. Jones

**Affiliations:** 1 Center for Sports Performance, George Mason University, Fairfax, VA USA; 2 Exercise & Sport Science Department, University of Wisconsin – La Crosse, La Crosse, WI USA; 3 Exercise & Sport Performance Laboratory, Kinesiology Department, Texas Christian University, Fort Worth, TX USA; 4 Exercise & Sport Nutrition Laboratory, Texas A&M University, College Station, TX USA; 5 Division of Health & Human Performance, George Mason University, 10890 George Mason Circle, MS 4E5, Manassas, VA 20110-2203 USA

**Keywords:** Nutritional supplementation, Dietary behaviors, NCAA student-athlete, Nutrient periodization, Survey

## Abstract

**Background:**

The purpose was to survey dietary habits (DH) and nutrient timing (NT) practices of baseball student-athletes (mean ± *SD;* 20.7 ± 1.4 yr.) from three NCAA Division I institutions, and examine the effect of a sports dietitian (SD) in regard to nutrition practices.

**Methods:**

Descriptive statistics and Pearson *X* ^2^ analyses were run. Responses on 10 DH and 5 NT items differed (*p* ≤ 0.10) between athletes who sought dietary planning from a SD (*n* = 36) versus those who consulted a strength and conditioning coach (SCC, *n* = 42).

**Results:**

In regard to DH items, the SD group found it easier to eat before activity (92% vs. 71%, *p* = 0.03), did not consume fast food (31% vs. 14%, *p* = 0.02), caffeinated beverages (57% vs. 46%, *p* = 0.02), or soda (56% vs. 37%, *p* = 0.10), prepared their own meals more often (86% vs. 73%, *p* = 0.07), and took daily multi-vitamins (56% vs. 32%, *p* = 0.02). The SCC group ate more at burger locations (21% vs. 6%, *p* = 0.02). In regard to NT items, the SD group ate breakfast before training/lifting sessions (67% vs. 37%, *p* = 0.02), and had post-workout nutrition options provided (61% vs. 27%, *p* = 0.01). The SCC group reported pre-competition meals of fast food (58% vs. 45%, *p* = 0.01), and sport coaches who were less aware of healthy food options (39% vs. 65%, *p* = 0.05).

**Conclusions:**

The SD is as a valuable asset to an intercollegiate athletics program. In the current study, athletes from the SD group consumed less high calorie/low nutrient dense items, ate before exercise, and consumed healthier options post-exercise. The presence of a SD was linked to provision of healthier food options during team trips. The evidence-based eating strategies and dietary plan provided by a SD may lead to improved performance and recovery.

## Background

Baseball is a sport consisting of short intense bouts of activity that is heavily reliant upon skill and technique [1]. Further, it has been reported that baseball energy system usage is approximately 90% anaerobic and 10% aerobic [1]. Since baseball is less aerobically taxing than many sports, nutrition may not be a primary focus of baseball coaches. However, baseball athletes participate in multiple games lasting upwards of three plus hours over the course of the collegiate season, which may have a substantial impact upon performance. Specifically, proper nutritional guidance in regard to dietary habits and nutrient timing practices is important for improving sport performance and ensuring proper recovery.

Nutritional needs and physiological stressors from the daily demands of training are elevated during the National Collegiate Athletic Association (NCAA) Division I baseball season, in which athletes play approximately 56 regular season games with the possibility of additional post-season games over a four to 5 month period [2]. The primary goal for these athletes should be one of nutritional guidance and education in order to ensure that chronic fatigue does not set in from weekly underfeeding and manifest itself negatively by the season’s end when competition levels peak. Baseball is also considered a sport in which student-athletes “Practice, play, eat, sleep, travel” [3] and this circle can reinforce inappropriate dietary behavior – particularly when the team is travelling for away games versus playing at home [4, 5].

Despite the growth in scientific knowledge surrounding proper nutrition, many athletes are still not achieving the recommended intake [6–9]. The sport dietitian (SD) or strength and conditioning coach (SCC) are frequently the main sources for disseminating dietary advice. Also, it has been previously reported in sports other than baseball that the SCC or athletic trainer are commonly utilized by athletes more often than the SD as the primary resource of nutritional information [10–12]. This is likely a result of student-athletes having limited access to a full-time RD [12] and more frequent interaction with their SCC or athletic trainer. The SD offers a pragmatic approach to nutrition education for athletes, while the SCC has consistent interaction with athletes throughout the training cycle and competitive season, but may not be current in regard to evidence-based knowledge of proper nutritional strategies. To date, the interaction of NCAA baseball athletes with the SD and/or SCC as a primary source of nutrition information has not been investigated.

Each baseball athlete has unique dietary requirements because nutrition needs and performance goals are rarely static and frequently vary by sport position. Traditionally baseball coaches group their athletes by position players and pitchers. However, sometimes position players are separated further into the groups of: catchers, corner infielders, middle infielders, and outfielders. This differentiation is important, as body mass index (BMI) differs between middle infielders compared to catchers and corner infielders with the latter positions having higher BMI [13]. With increased BMI comes improved offensive capabilities, such as runs batted in, homeruns, and slugging percentage [14]. Further, approaches to strength and conditioning programming often vary as each baseball sport position may require different skill sets and physical characteristics with noted differences reported in VO_2max_ and speed to first base [15, 16]. These differences create a heterogeneous base of athletes with different physiological demands per position, with an additional noted difference in energy burned per minute between pitchers and position players [3, 17].

The current study was designed to provide a more comprehensive view of dietary habit differences in NCAA Division I baseball student-athletes by assessing a wide spectrum of nutritional practices, ranging from nutrient periodization, which is the deliberate manipulation of macronutrient intake to match training goals, and enhance performance [18], to pre-workout and post-workout dietary habits. The primary aim of the present study was to examine differences between using a SD or a SCC as the main source of nutrition information on the dietary habits and practices of NCAA Division I baseball players. The secondary aim was to assess how baseball sport position may influence dietary habits and practices. It was hypothesized that there would be differences in the dietary habits and nutrient practices between those baseball athletes with access to a SD and those without access. Further, we hypothesized baseball sport position would influence dietary habits and practices.

## Methods

### Overview

This descriptive research study employed a cross-sectional survey designed to assess dietary habits and nutrient timing practices in NCAA Division I baseball student-athletes. A survey questionnaire was designed in a similar fashion to ones previously used to assess the dietary eating habits of NCAA collegiate athletes [19]. The use of the current survey allowed for the assessment of specific nutrition habits, which included hydration and supplement use, as well as nutrient periodization strategies across three baseball programs at the NCAA Division I level.

### Subjects

Subjects were a total of 99 male (mean ± SD: age = 20.7 ± 1.4 yr.; age range = 18–23 yr.) NCAA Division I baseball student-athletes consisting of freshmen (*n* = 19), sophomores (*n* = 18), juniors (*n* = 34), seniors (*n* = 26), and graduate students (*n* = 2), from three universities in three athletic conferences ((Atlantic 10 (*n* = 31), Atlantic Coast Conference (*n* = 32), Conference USA (*n* = 36)). The number of athletes on athletic scholarship was consistent across the three participating institutions. All athletes were medically cleared for intercollegiate athletic participation, had the investigative procedures explained to them beforehand, and signed an institutionally approved consent form to participate. The Institutional Review Board for Human Subjects at each participating institution approved all procedures. Subject descriptive data are detailed in Table 1.

**Table 1:** Characteristics of athletes by participating university

	Age (year)	On-campus Housing	Sport Position: Catcher	Sport Position: Pitcher	Sport Position: Corner Infield	Sport Position: Middle Infield	Sport Position: Outfield	Diet Provider: Sport Dietitian	Diet Provider: Strength Coach	Diet Provider: Other/No response
University 1 (*n* = 31)	20.7 ± 1.4	16	3	16	4	5	3	19	0	7/5
University 2 (*n* = 32)	20.3 ± 1.5	0	3	13	2	7	6	17	9	4/2
University 3 (*n* = 36)	21.1 ± 1.4	11	3	15	4	7	7	0	33	2/1

Age values are mean ± SD. Other characteristic values are subject number (n). No response indicates the number who left the item unanswered. Corner infield positions consist of 1st and 3rd base. Middle infield position consists of 2nd base and shortstop

### Sports dietitian

Two of the three institutions employed a full-time sports dietitian (SD). For the current study, a SD was defined as a registered dietitian (RD) with the Board Certified Specialist in Sports Dietetics (CSSD) from the Academy of Nutrition and Dietetics. Responsibilities of the SD included working with individual athletes, teams, coaches, and other athletic staff members to provide nutrition education and counseling with the primary aim being that of improving athlete performance and recovery. Information was also distributed through general outreach efforts by the SD, such as educational bulletin boards, athletics/sports nutrition website content, social media, sports nutrition lectures, and informal interaction with the athletes and athletic staff.

### Strength and conditioning coach

All three institutions employed a strength and conditioning coach (SCC) who was assigned to work with baseball. All SCCs in the current study held the National Strength and Conditioning Association’s Certified Strength and Conditioning Specialist credential (NSCA-CSCS). However, none held the Certified Sports Nutritionist from the International Society of Sports Nutrition credential (CISSN), which is a professional accreditation that requires practitioners to posses a basic knowledge of sports nutrition without necessarily holding the RD credential. Responsibilities of the SCC included resistance training program design and implementation throughout the academic year, attendance at selected sport practices, and varying degrees of travel with the team during the season.

### Procedures

In a prior study, the authors designed the current survey questionnaire in order to assess the dietary habits of NCAA Division I athletes (20), which was developed based upon one previously used to assess the dietary eating habits of NCAA Division III athletes [19]. Procedures were followed to establish content validity. Initially, a qualitative researcher with content knowledge of sport nutrition reviewed the survey. Suggestions in regard to placement and wording of certain questions were incorporated into the second version. Next, two SDs from the two participating institutions reviewed the instrument. Suggestions that were added into the third version included a separate section on hydration and representative pictures of serving sizes. Finally, the survey was piloted with a group (*n* = 6) of athletic trainers, graduate assistant coaches, and strength coaches from the involved universities. Suggestions provided by the aforementioned group related primarily to phrasing of specific questions, and were taken into account during the completion of the fourth version, which was the final draft. No participants in the pilot study served as subjects in the current study.

The survey questionnaire consisted of 62 total questions distributed over nine sections. The nine sections were represented in the following order: sport participation, general dietary habits, breakfast, hydration, nutritional supplements, post-workout nutrition, nutrition during team trips, nutrient periodization, and demographic information. There were 25 close-ended, 22 interval, 7 multiple choice, and 8 open-ended questions. Open-ended questions were asked in regard to: demographics (*n* = 2), eating habit changes with training season (*n* = 2), listing of breakfast foods (*n* = 1), listing of supplements currently using (*n* = 1), and sports participation (*n* = 2).

### Data collection

The same researchers administered the survey questionnaires during scheduled testing sessions. First, athletes read and signed the informed consent form. Next, researchers reviewed the survey’s instructions with the subjects, and remained throughout the testing sessions in order to answer questions. Athletes were provided a pencil and a survey, sat apart from each other, and no talking or leaving their seats was permitted. Upon completion, subjects placed the survey questionnaire into an envelope. All survey questionnaires were anonymous. The only identifying information consisted of age, gender, sport, and university name. Sport coaches were not present during data collection. Athletes were surveyed prior to a strength training session. There was no time limit and the total time for completion of the survey ranged from 15 to 20 min depending upon the individual.

### Data analysis

The data were analyzed to present descriptive data sorted by the athlete’s source of nutritional information (i.e., SD, SCC, other). Data analysis consisted of descriptive statistics and 2-way Pearson X^2^ analyses. Alpha was set *p* ≤ 0.10 for statistical significance, which is often used with self-rating and survey measures. All data were analyzed using SPSS V.22 (IBM Corporation; Armonk, NY).

## Results

All three institutions employed a full-time SCC, but only two employed a full-time SD. Of the 99 total survey respondents, when asked who was in charge of implementing/directing their sport dietary plan, 36 answered SD, 42 answered SCC, and 21 selected “other” or did not answer. Responses on 10 dietary habit and 5 nutrient timing items differed (*p* ≤ 0.10) between athletes who sought dietary planning from a SD (*n* = 36) versus a strength and conditioning coach (SCC, *n* = 42). A majority of baseball athletes from Schools 1 (61%) and 2 (53%) reported working with the SD for individual performance nutrition (sport dietary) planning assistance (Table 1).

**Fig. 1:**
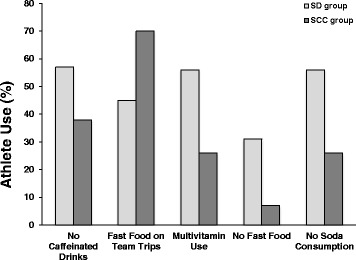
Selected dietary habits of the SD and SCC groups

Significant differences were observed in dietary habit items between baseball athletes who worked with a SD and those who did not. Figure 1 represents the response rate in regard to selected dietary habits of the SD and SCC groups. A greater number of athletes from the SD group reported never consuming fast food (31% vs. 7%, *X* ^2^ = 18.57, *p* = 0.02), caffeinated beverages during the weekday (Monday-Friday) (57% vs. 38%, *X* ^2^ = 18.27, *p* = 0.02), or soda on a weekend day (Saturday/Sunday) (50% vs. 26%, *X* ^2^ = 10.56, *p* = 0.08). Further, the SD group was more likely to take a daily multi-vitamin (56% vs. 26%, *X* ^2^ = 17.78 *p* = 0.02) and consume fast food less frequently on team trips (45% vs. 70%, *X* ^2^ = 9.984 *p* = 0.01). Fast food was defined as food that can be prepared and served quickly without prior seating by a host or server.

Additional differences in dietary habits between groups included: 1) the SCC group ate more frequently at burger locations (21% vs. 6%, *X* ^2^ = 12.28 *p* = 0.02), and 2) the SD group was more likely to have meals preplanned by the SD when travelling on team trips (48% vs. 13%, *X* ^2^ = 11.37 *p* = 0.01).

**Fig. 2:**
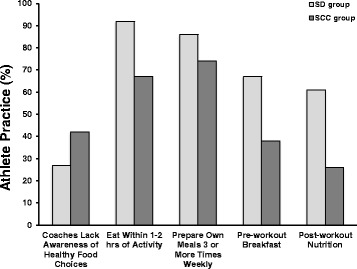
Nutrient timing practices of the SD and SCC groups

Figure 2 represents the significant differences in the response rate of selected nutrient timing issues of the SD and SCC groups. The SCC group reported their sport coaches to be less aware of healthy food choices for the team when travelling (42% vs. 27%, *X* ^2^ = 9.67, *p* = 0.05). The SD group found it easier to eat within 1–2 h of activity (92% vs. 67%, *X* ^2^ = 7.32, *p* = 0.03), was more likely to have a pre-workout breakfast (67% vs. 38%, *X* ^2^ = 8.03, *p* = 0.02) as well as post-workout nutrition options provided (61% vs. 26%, *X* ^2^ = 9.91, *p* = 0.01). The SD group was also more likely to prepare ≥3 meals on their own each week (86% vs. 74%, *X* ^2^ = 19.96, *p* = 0.07).

Protein shakes and bars were popular options for post-workout nutrition in both the SD (56%) and SCC (46%) groups; however, the SCC group was more likely to have a post exercise meal that comprised of food items such as eggs (51% vs. 22%, *X* ^2^ = 8.15, *p* = 0.02) and fruit or fruit juice (37% vs. 11%, *X* ^2^ = 7.02, *p* = 0.03). Also, the SCC group was more likely to eat a complete meal within 1-h post-exercise (95% vs. 78%, *X* ^2^ = 16.51, *p* = 0.01).

As shown in Table 2, use of a protein supplement was most popular among supplement users (60.98%) and remained most popular regardless of primary nutrition information source. Supplement users also favored multivitamins and fish oil (41.46 and 21.95%, respectively). Other supplements of note were creatine (14.63%), individual vitamins (12.20%), and energy supplements (9.76%).

**Table 2:** Prevalence and type of supplement usage

Supplement	Prevalence among all survey respondents (*n* = 99)	Prevalence among reported supplement users (*n* = 41)
Protein	25.25%	60.98%
Multivitamin	17.17%	41.46%
Fish Oil	9.09%	21.95%
Creatine	6.06%	14.63%
Individual Vitamins	5.05%	12.20%
Other	4.04%	7.32%
Energy Supplement	3.03%	9.76%

A few differences were observed among position groups. Middle infielders (i.e., 2nd base, shortstop) found it easier to eat within 1–2 h pre-exercise ((59% vs. 29% (average of other position groups), *X* ^2^ = 10.82, *p* = 0.03)), and were more likely to be taking a supplement ((74% vs. 41% (average of other position groups), *X* ^2^ = 12.42, *p* = 0.01)). Pitchers consumed fewer caffeinated beverages than other position groups (((93% vs. 50% (average of other position groups), *X* ^2^ = 31.94, *p* = 0.04)).

## Discussion

Our primary aim was to examine differences between using a SD or a SCC as the main source of nutrition information on the dietary habits and practices of NCAA Division I baseball players. The secondary aim was to assess how baseball sport position may influence dietary habits and practices.

Prior research, including the results of the present study, has shown that access to a registered dietitian or SD can help NCAA athletes increase adherence to performance nutrition principals [20, 21]. However, time constraints can prevent SDs from providing proper nutrition education to all athletes under their purview, and many NCAA institutions have no SD available due to financial limitations. At present, 88 full-time SDs are employed at 61 schools in major college conferences in the United States – many of whom work as the sole SD, which may require them to be responsible for >600 student athletes [22]. Due to limited SD access and availability, it is not uncommon for student-athletes to seek out SCCs for nutrition guidance. Previous publications have reported that 16.2 to 28% of college athletes will use SCCs as their primary source of nutrition information [10–12, 23]. Additionally, SCCs will interact with athletes multiple times during a week, providing opportunities to help reinforce best performance nutrition practices. Therefore, it is desirable for SCCs to have an adequate understanding of evidence-based nutrition practices for optimizing sports performance.

Two studies have attempted to assess the nutrition knowledge level of SCCs [12, 24]. Torres-McGhee et al. evaluated sports nutrition knowledge of 71 SCCs using 20 multiple choice questions answered via a voluntary survey [12]. Using a cutoff of 75% to indicate “adequate nutrition knowledge”, 83.1% of respondents met or exceeded these criteria while 16.9% showed inadequate knowledge. Scores were higher in questions regarding supplements and performance (90.4%), weight management and eating disorders (80.3%), and hydration (79.4%). Knowledge of micronutrients and macronutrients scored the lowest (76.1%). Smith-Rockwell et al. assessed 10 SCCs via a voluntary 20-question survey (29). Participants correctly responded 80% of the time. Due to the limited sample sizes, the brief nature of the questionnaires, and the potential for volunteer bias, it is difficult to generalize these results. However, they preliminarily suggest that SCCs may possess adequate knowledge of general sports nutrition. Further, the results of the current study may help indicate areas in which it can be advantageous for SCCs to increase their performance nutrition knowledge. Nutrient timing, food quality, supplement use, and alcohol consumption have been identified as potential areas of interest when educating student-athletes on best performance nutrition practices.

Timing of nutrient intake is an essential component to support training, reduce fatigue, optimize recovery, and assist in maintaining body composition [25]. Baseball players should be advised to employ eating strategies that provide adequate pre-, post-, and during- workout nutrition. In the current study, baseball players, who used SCCs as their primary nutrition information source, experienced favorable compliance to post-workout recommendations. In fact, they were more likely to consume food within a 1-h window after training, or competition (95%). However, only 67% indicated it was easy for them to eat 1–2 h pre-exercise, 38% consistently ate pre-exercise breakfast, and 26% were provided with post-workout nutrition options. Providing post-workout dietary options may not be financially or logistically feasible for some. Since compliance to post-workout food consumption within 1 h of training is already high, SCCs might consider verbal reminders as well as posting signage in regard to proper post-workout nutrition guidelines in order to encourage greater athlete adherence to appropriate dietary intake.

Inappropriate fueling strategies leading to hunger or dehydration is another area of concern that SCCs can help to address. Within the SCC group, 66% of baseball athletes reported experiencing an episode of hunger during training, practice, or competition compared to 72% in the SD group. Within the SCC group 22% reported having suffered negative effects due to dehydration while the SD group reported 18%. Although the frequency of hunger episodes or dehydration was not obtained, experiencing either during exercise is indicative of poor fueling. A consistent lack of appropriate dietary planning can lead to chronic performance decreases, muscle protein catabolism, and impaired recovery status [25]. Athletes should be encouraged to follow recommendations to consume a pre-exercise meal [25] and to replace lost bodily fluids [25, 26].

Convenience and low cost are primary contributors that drive fast food consumption among college students [27, 28]. Data from our study showed that SCC group ate fast food with greater frequency throughout a 7-day week than the SD group. On the upper end of consumption, the SCC group saw 17% of athletes consuming 5 to 8 fast food meals in a week compared to 3% in the SD group. Additionally, reports of fast food being provided prior to a practice or competition while on team trips were high for both conditions (SCC 70% vs. SD 45.45%). Prior research has indicated that average fast food consumption across male and female collegiate teams to be between 13.69 [20] and 15% (30%), but baseball was not represented. A survey of baseball players (*n* = 25) reported a high interest in learning about healthier fast-food alternatives [29]. It is likely baseball players would be receptive to this information delivered through a variety of educational methods. The survey also reported baseball players had equally high preferences for receiving nutrition education through group sessions, individual counseling, newsletters, study modules, computer training, and conferences [29]. Modestly higher interest was reported for nutrition education through academic courses or from nutrition graduate students [29]. SCCs could employ any number of these methods to communicate fast food alternatives.

In regard to supplement usage, 42% of baseball players reported taking a supplement (SCC 37% vs. SD 53%). This is an overall lower incidence of supplement use than previously reported in collegiate athletes [10, 30, 31]. When compared to NCAA survey data on the use of dietary supplements among baseball players, we found comparable use of creatine (17% vs. 18.8% NCAA) and lower use of protein (25% vs. 58.9% NCAA) and multivitamins (6% vs. 28.1% NCAA) [32]. Inadvertent ingestion of a supplement containing or contaminated with NCAA impermissible substances can lead to temporary or permanent loss of NCAA eligibility [33]. Since prior research has shown athletes’ knowledge to be low in areas of supplement use [12, 21, 31] and safety [21, 31], SCCs can be a valuable resource on the risks and efficacy of supplements.

Alcohol consumption during periods of heavy training or competition, as commonly seen during the pre- and in- season, may cause dehydration and increase recovery time [25]. The amount and frequency of alcohol ingestion were comparable between the SCC and SD groups. A 2014 NCAA report on substance use trends in NCAA athletes found that 92% reported either never consuming alcohol or doing so ≤2 days per week [32]. A similar rate of 86% was observed in our study. The 2014 NCAA report also found that 49.3% drank during their competitive season – a modestly higher percentage than the 40% observed in the current study. An earlier 2012 NCAA report found 49% of athletes were at risk of binge drinking [34]. The National Institute on Alcohol Abuse and Alcoholism define binge drinking as “a pattern of drinking that brings blood alcohol concentration levels to 0.08 g/dL… [typically occurring] after 4 drinks for women and 5 drinks for men—in about 2 hours” [35]. The response rate of the baseball athletes in the current study places 52% at risk for binge drinking behaviors.

Differences in dietary habits were noted across sport position groups. Middle infielders found it easier to eat pre-exercise and were more likely to be taking a supplement. Of the middle infielders surveyed, 63% were using a protein supplement. Multivitamins, fish oil, and creatine were also popular supplements for this sport position group. It is possible that middle infielders were attempting to use supplements to support weight gain goals as a higher BMI has been associated with improved offensive measures when examining performance data over multiple decades [14, 36]. Further, a significant relationship has been reported between home runs and BMI with home runs per season and BMI reaching their highest levels in the most recent decade [36].

While the strengths of our study included a large, homogenous sample size of baseball players and a high survey response rate, there remain limitations of note. First, survey data were grouped and analyzed based upon whom the baseball athletes identified as their primary source for individual performance nutrition (sport dietary) planning assistance. Second, as with most survey research, limitations include susceptibility to recall bias of survey questions, under- or over-reporting by survey respondents, and the assumption that respondents are answering questions honestly.. Lastly, there was unequal access to a SD across institutions as only two of the three schools surveyed employed a full-time SD; therefore, it is possible that some of the differences or similarities observed may have been influenced by outreach efforts or general interactions between the SDs and SCCs, athletes, or other sports performance staff members. Further, a team’s funding level may affect an athlete’s use of/or access to resources that may improve performance, such as supplements and food. Teams with better funding may be provided with a more complete array of sports nutrition services.

## Conclusions

The SD is as a valuable asset to an intercollegiate athletics program. However, in institutions with limited financial resources, the SCC may serve as the athlete’s primary knowledge source of nutrition as it relates to sports performance. Therefore, it is recommended that SCCs seek ways to improve their knowledge of nutrient timing, hydration practices, as well as methods to decrease alcohol consumption and provide convenient, healthy food options to their athletes. When plausible, it is recommended that SCCs form collaborative relationships with SDs. Such relationships can be beneficial in the event that a situation arises which falls outside of the SCC’s scope of practice.

## Abbreviations

BMI: Body mass index
DH: Dietary habits
NCAA: National Collegiate Athletic Association
NT: Nutrient timing
SCC: Strength and conditioning coach
SD: Sports dietitian
